# Completion Axillary Lymph Node Dissection (cALND) in Residual Axillary Disease After Modified Radical Mastectomy

**DOI:** 10.7759/cureus.117391

**Published:** 2026-10-03

**Authors:** Shantanu Tyagi, Mohim Thakur, Mridul Khanna, Anshul Sharma, Anilkumar Chongtham

**Affiliations:** 1 General Surgery, All India Institute of Medical Sciences, Bilaspur, Bilaspur, IND; 2 Medical Oncology, All India Institute of Medical Sciences, Bilaspur, Bilaspur, IND; 3 Nuclear Medicine, All India Institute of Medical Sciences, Bilaspur, Bilaspur, IND; 4 Surgery, All India Institute of Medical Sciences, Rishikesh, Rishikesh, IND

**Keywords:** completion axillary lymph node dissection, metastatic breast cancer, methylene blue dye, modified radical mastectomy, mrm, radiotherapy, residual axillary disease, sentinel lymph node

## Abstract

Residual axillary disease after an incomplete axillary lymph node dissection in breast cancer poses a management challenge. We report a 46-year-old female with left breast invasive ductal carcinoma who presented two months after modified radical mastectomy with bulky residual axillary nodes. PET-CT revealed metabolically active nodes suspicious for metastasis. After multidisciplinary discussion, completion axillary lymph node dissection (cALND) was performed, yielding two metastatic nodes out of six. The postoperative course was uneventful, and the patient proceeded to systemic therapy. For limited residual disease, regional nodal irradiation plus systemic therapy may achieve comparable local control with reduced morbidity, whereas bulky disease may justify surgical clearance for accurate staging and local control. This case highlights the role of cALND in young patients with significant residual nodal disease, emphasizing individualized, multidisciplinary decision-making based on disease burden, radiation quality, and systemic therapy.

## Introduction

Breast cancer is significantly rising worldwide. Modified radical mastectomy (MRM) or breast-conserving surgery (BCS) with systemic therapy remains the cornerstone for treatment of breast cancer. Axillary lymph node dissection (ALND) is a routine component of the surgical management of breast cancer. Completion axillary lymph node dissection (cALND) is also done if, intraoperatively, sentinel nodes harbor malignancy. Surgery for breast cancer is performed by surgeons working in centers ranging from small-volume district hospitals to high-end tertiary hospitals in India. There is a possibility that inadequate ALND may be performed, leading to residual disease in the axilla that is diagnosed later during outpatient follow-up.

The management plan of residual nodal disease diagnosed after a formal MRM is debatable. Previous literature suggests systemic therapy as optimum for such patients, offering comparable local recurrence and overall survival rates. cALND for such residual nodal disease has not been described routinely in the literature [1]. Herein, we discuss the management of such a case where cALND was suggested after a multidisciplinary tumor board discussion in view of the young age and bulky residual axillary disease.

## Case presentation

A 46-year-old female was referred to the outpatient clinic of our department as a follow-up case of carcinoma of the left breast, status post left breast MRM two months ago (operated in an outside center) for a cT3N1Mx disease in the left breast, which was biopsy-proven to be invasive ductal carcinoma of the breast that was estrogen receptor (ER)- and progesterone receptor (PR)-negative and human epidermal growth factor receptor 2 (HER2)-positive. Preoperative radiological investigations were not available from the outside center (we acknowledge this limitation). Postoperative histopathology showed that nine lymph nodes were isolated, one of which showed a tumor deposit (1/9). The patient came with complaints of pain in the left forearm and axilla. The pain was dull, aching, intermittent, and progressive in nature, initially limited to the elbow, now radiating up to the digits. It was not associated with any edema or skin changes. There was no history of any numbness or tingling sensation in the left upper limb or any backache. The patient was a known case of rheumatoid arthritis, on oral methotrexate for two years. Pain was not limited to joints. She did not suffer from any other comorbidity. There was no family history of any malignancy in immediate first-degree relatives. On examination, the patient was afebrile with stable vitals. Breast examination depicted a healed scar of the left breast post MRM. The lateral aspect of the scar revealed changes similar to a peau d’orange appearance in the skin. There was a 3 × 3 cm mobile lump in the left axilla, suggestive of lymphadenopathy. There was no palpable lump in the contralateral breast, axilla, or neck. Also, there were no sensorimotor disturbances in the left upper limb. Abdominal examination was unremarkable.

The patient was admitted to our department and further evaluated. PET-CT showed metabolically active (maximum standardized uptake value (SUVmax) = 7) left bulky axillary lymph nodes with partially obliterated fatty hilum and thickened cortex, the largest measuring 1.5 cm, suspicious for residual metastasis; no definite scan evidence of metabolically active malignant disease was identified elsewhere in the surveyed region (Figure 1).

**Figure 1:**
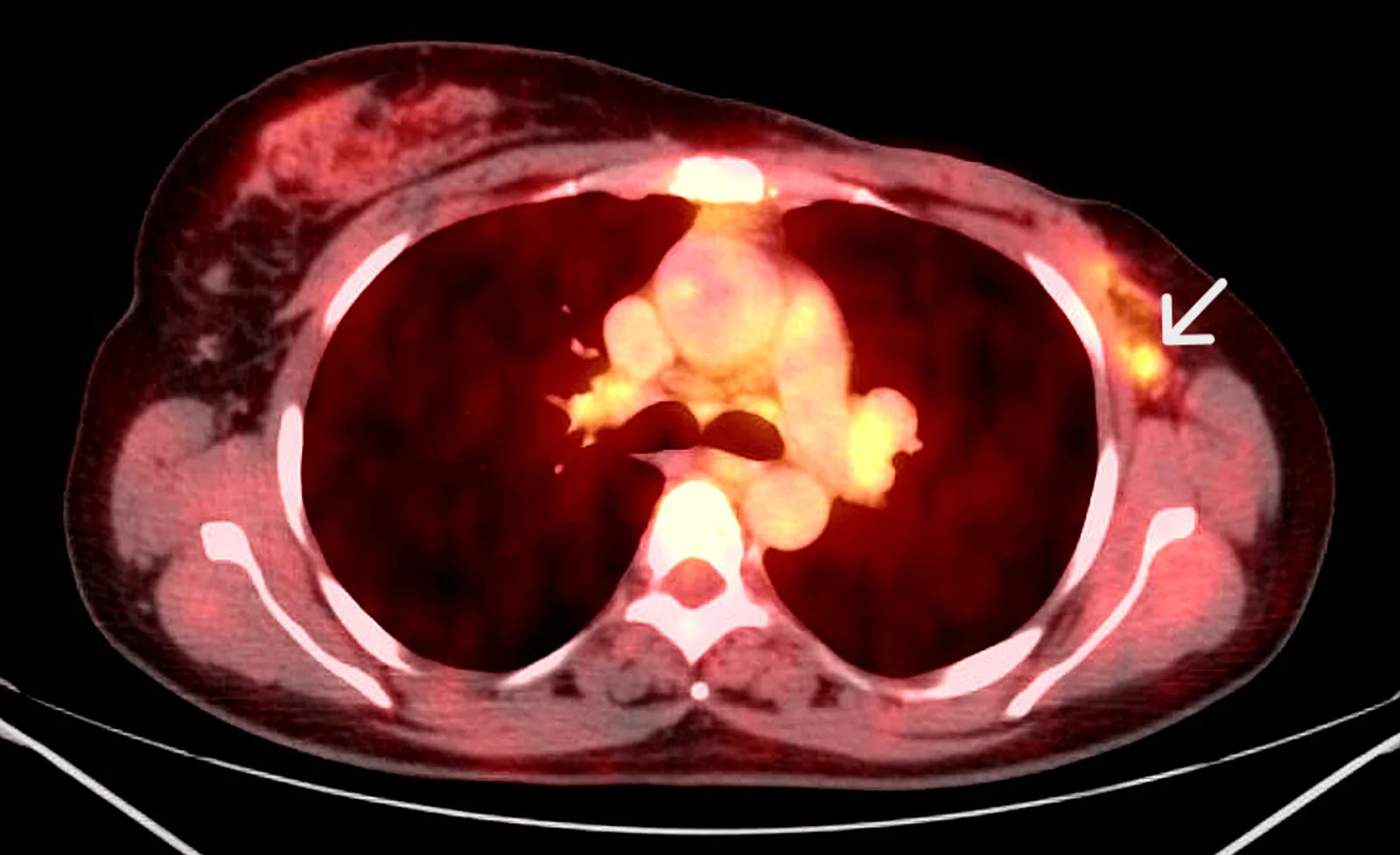
PET-CT image showing a fluorodeoxyglucose (FDG)-avid nodal mass (white arrow), with the largest lymph node measuring 1.5 cm, in the left axilla (maximum standardized uptake value = 7), suggestive of residual nodal metastasis.

Given these findings, the case was discussed in a multidisciplinary tumor board, including a medical oncologist, radiation oncologist, surgical oncologist, and general surgery team, which suggested cALND, followed by systemic therapy as the further plan of action, considering the young age of the patient and bulky nodal disease, including limited patient follow-up as she belonged to remote areas of North India. Therefore, cALND was planned to reduce the tumor burden.

Intraoperatively, methylene blue dye was injected around the previous scar near the left axilla. An incision was made along the left axilla, and dye uptake was noted within the residual axillary fat pad (Figure 2). Multiple matted lymph nodes were identified up to the axillary vein. The axillary fat pad was dissected from the axillary vein to the angular vein distally, preserving the thoracodorsal bundle and serratus anterior nerve (Figure 2).

**Figure 2:**
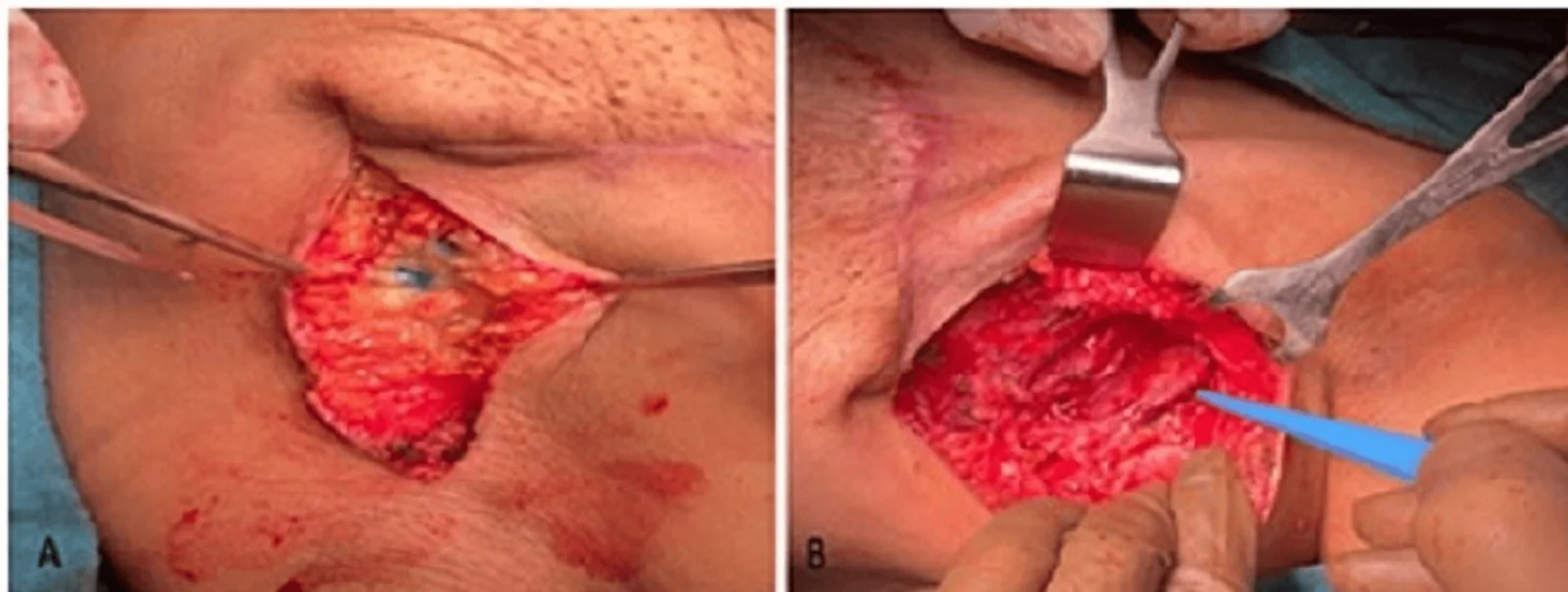
(A) Intraoperative picture showing methylene blue uptake of the lymph node and (B) intraoperative image showing preserved thoracodorsal pedicle during completion axillary lymph node dissection (cALND).

All lymph node masses were removed en bloc and sent for histopathological examination. Histopathological examination showed two out of six lymph nodes (2/6) with metastatic tumor deposits (Figure 3).

**Figure 3:**
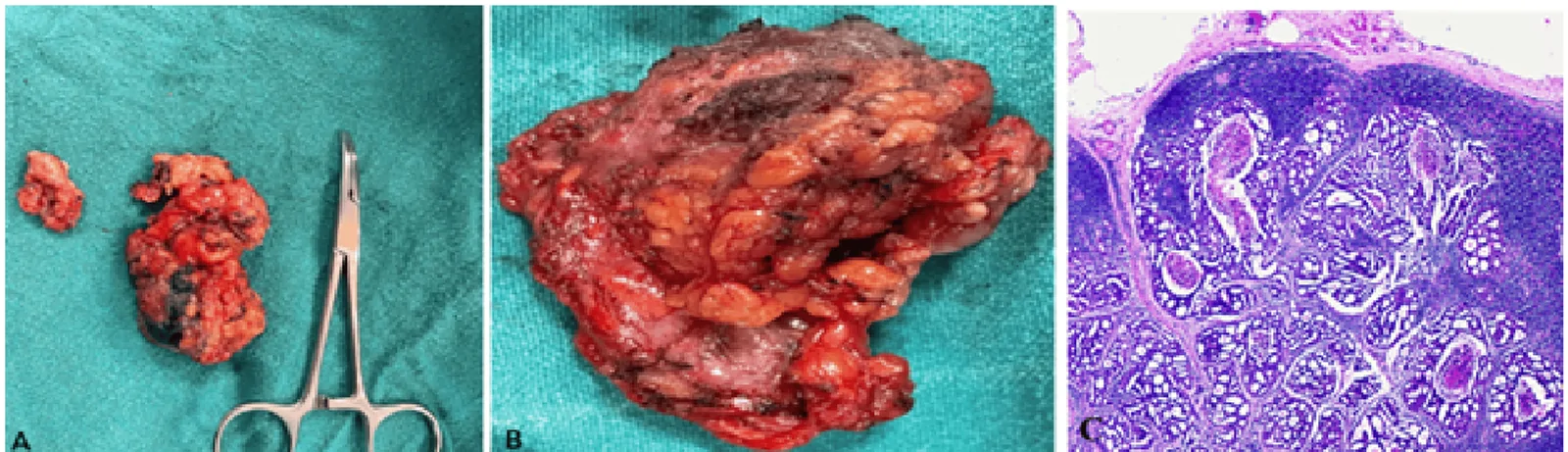
(A) Gross specimen of the lymph node mass removed during axillary lymph node dissection. (B) Cut section of en bloc lymph node mass. (C) Microscopic appearance of the metastatic lymph node (100x).

The remaining four lymph nodes showed reactive hyperplasia. The postoperative course was uneventful. The patient was discharged on postoperative day (POD) 3, and a closed suction drain was removed on POD 10 during a follow-up visit. The patient followed up with the oncology team for systemic therapy thereafter. The patient received trastuzumab-based systemic therapy (ado-trastuzumab emtansine (T-DM1), 3.6 mg/kg IV administered once every three weeks) for 14 weeks, starting three weeks postoperatively. Postoperative lymphedema status and dull aching pain improved gradually (visual analog scale score was 2 at 1.5 months postoperatively and on systemic therapy), and currently the patient is doing well with regular follow-up (currently three-monthly) with both the oncology and general surgery departments.

## Discussion

Residual nodal disease after a possible incomplete ALND presents two main, guideline-concordant options: proceed to cALND or treat the undissected axilla with regional nodal irradiation, alongside systemic therapy tailored to tumor biology. Over the past decade, randomized and contemporary data have driven a broad de-escalation of axillary surgery in carefully selected patients, centering on oncologic equipoise between surgery and radiation for local control, and the primacy of systemic therapy for survival.

The AMAROS randomized trial provides the clearest comparator between ALND and axillary radiotherapy (ART) for patients with a positive sentinel node after upfront surgery. Its 10-year analysis showed very low axillary recurrence rate (ARR) with either strategy (10-year ARR ~0.9% after ALND vs. ~1.8% after ART) and no differences in disease-free or overall survival; critically, lymphedema was roughly doubled after ALND (24.5% vs. 11.9%) [2]. Although AMAROS enrolled patients with a positive sentinel node rather than explicitly inadequate ALND, the biological problem - residual microscopic axillary disease - overlaps. Thus, in many postoperative scenarios where residual disease risk exists, ART achieves excellent control with less arm morbidity.

More recently, the randomized SENOMAC trial broadened the paradigm by including patients undergoing mastectomy, T3 tumors, and microscopic extracapsular extension. Omission of cALND (i.e., sentinel lymph node biopsy (SLNB) alone) was non-inferior to completion dissection for overall survival; most patients received nodal radiotherapy (RT) per national standards, again supporting that radiation plus systemic therapy can replace more extensive axillary surgery without compromising outcomes [3]. Reflecting this evidence base, the 2025 American Society of Clinical Oncology (ASCO) SLNB guideline update and contemporary reviews endorse selective omission of cALND when nodal disease burden is limited and appropriate systemic and radiation therapy will be delivered [4,5].

Where does this leave a patient with residual axillary disease after an inadequate ALND? Decision-making hinges on three elements: (i) extent and biology of residual disease (macroscopic residual nodes, matted/extranodal extension (ENE) vs. presumed microscopic); (ii) planned regional nodal irradiation quality/coverage; and (iii) systemic therapy intensity. If there is bulky residual nodal disease, grossly involved nodes, or an inability to deliver comprehensive nodal RT, cALND remains reasonable to ensure local control and accurate staging for adjuvant escalation. Conversely, if residual disease is likely microscopic and high-quality ART is planned, the weight of AMAROS/SENOMAC-aligned data favors ART over cALND to minimize lymphedema without sacrificing control.

The neoadjuvant setting deserves special mention because many patients undergo systemic therapy first and may have ypN+ disease. Here, practice is in flux. Until definitive randomized results are reported, many guidelines still consider ALND standard for residual nodal disease after neoadjuvant therapy; however, ongoing phase III work (Alliance A011202) is directly comparing ALND to axillary radiation in patients with positive sentinel nodes after neoadjuvant chemotherapy [6]. Contemporary expert reviews emphasize that, pending these results, omission of ALND for ypN+ disease should be judicious, ideally within trials, whereas ART is a rational de-escalation strategy when nodal burden is limited and comprehensive regional nodal irradiation (RNI) will be delivered [5,7,8].

Finally, modern systemic therapy (e.g., HER2-directed regimens, capecitabine for triple-negative breast cancer (TNBC), CDK4/6 inhibitors for hormone receptor (HR)-positive high-risk disease) substantially lowers distant and regional failure risks, further reducing any marginal benefit from added axillary surgery while not mitigating surgery-related morbidity. Accordingly, adjuvant therapy should be optimized per phenotype and residual-disease risk irrespective of whether cALND or ART is chosen [4,5].

Limitations

Unavailability of preoperative radiological records from the outside center where the patient was operated is a limitation of the study.

## Conclusions

In summary, for residual axillary disease after ALND, cALND improves pathologic staging but carries a higher lymphedema risk. ART offers comparable long-term control with less morbidity when residual disease is limited. Our case highlights the role of cALND, especially in patients who are young with bulky residual disease in the axilla necessitating surgical clearance to ensure local control and accurate pathological staging. A nuanced, multidisciplinary choice balancing disease burden and quality of radiation/systemic therapy is appropriate.
